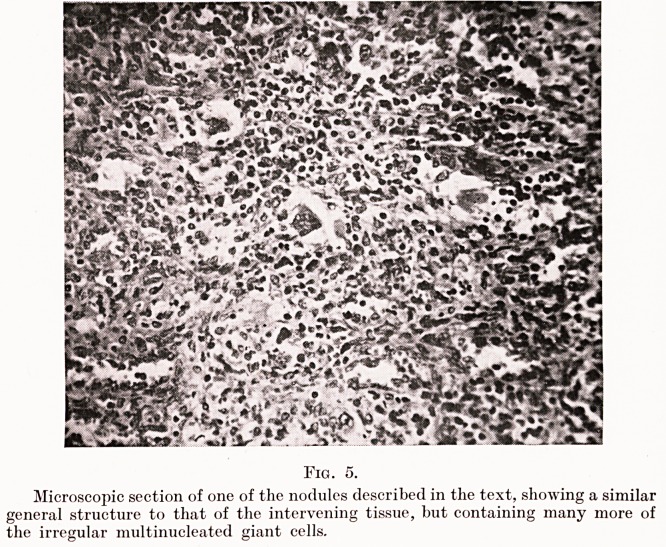# A Case of Primary Sarcoma of the Spleen

**Published:** 1929

**Authors:** Arthur L. Taylor

**Affiliations:** Pathologist, Bristol General Hospital


### A CASE OF PRIMARY SARCOMA OF THE SPLEEN.

BY

ARTHUR L. TAYLOR, M.D.,
*Pathologist, Bristol General Hospital.*

---

MALIGNANT disease of the spleen is a rare condition. This very vascular organ might well be expected to show deposits of growth in cases where there is widespread dissemination of malignant cells by the bloodstream, yet such deposits are extremely seldom seen, a fact which is without doubt related to the destructive function exercised by the splenic pulp. Primary growths, though rather more common than secondary, are still to be regarded as pathological rarities. For this reason the following case of primary sarcoma recently encountered at the Bristol General Hospital is considered worthy of record.

*Case Report.*—On 19th January the patient, a woman of 54 years, attended the Casualty Department of the Hospital complaining of severe epigastric pain which had started quite suddenly three days before and had lasted continuously ever since. For the last month she had suffered from general weakness and some shortness of breath on exertion, but there had been no previous acute attacks. The pain was griping in character, but did not cause vomiting ; there had been no hæmatemesis or melæna. Preliminary examination revealed an enormously enlarged spleen, and the patient was admitted forthwith for full investigation.

On admission the patient was a thin, pale woman with a slightly icteric tinge of the skin and conjunctiva. Temperature 99°, pulse 89, respirations 20, blood-pressure 120. Careful examination failed to reveal any gross organic disease of the heart, lungs, alimentary system or of the central nervous system. In the abdomen a large swelling, obviously the spleen, was found extending well across the middle line and almost as low as the pubes. The anterior notch could be well made out, and the surface, easily palpated through the wasted abdominal wall, felt nodular to the touch. There was no increase in size of the liver, and no ascites or œdema ; the lymph glands throughout the body showed no enlargement.

The case, then, was one of massive splenic enlargement associated with evident anæmia, and an interesting problem in differential diagnosis was presented.

A differential blood-count gave the following results : Red cells, 3,480,000 per cmm. ; hæmoglobin, 40 per cent. ; colour index, 0·58 per cent. ; leucocytes, 7000 per cmm. ; neutrophile polymorphs, 67·5 per cent. ; eosinophile polymorphs, 2·0 per cent. ; basophile polymorphs, 1·0 per cent. ; lymphocytes, 25·5 per cent. ; large hyalines, 4·0 per cent.

These findings indicate an anæmia of secondary type, and served at once to exclude polycythæmia, pernicious anæmia and leukæmia, as well as any of the acute infections in which a leucocytosis or leucopenia is to be found. Considering the possibility of lymphadenoma, the differential leucocyte count was of importance, since in this condition there is commonly a considerable relative increase in large hyaline cells. No such increase was found ; moreover, the absence of lymph gland enlargement and the enormous size of the spleen were sufficient in this case to negative a diagnosis of Hodgkin's disease.

A further possibility to consider was the splenomegaly associated with acholuric jaundice. This condition, in which the spleen sometimes reaches enormous proportions, is characterized by two peculiar features ; an increased fragility of the red cells and the appearance of colloidal bilirubin in the blood-stream. In the present case the red cell fragility was normal, and Van den Bergh's reaction, both direct and indirect, was negative.

Gaucher's disease, in which the splenic enlargement is due to extreme lipoid infiltration of the endothelial cells of the splenic pulp, was put out of court because in this condition a similar lipoid infiltration of the liver and lymph glands produces an associated enlargement of these organs.

There was, then, remaining that ill - defined type of splenomegaly referred to as " splenic anæmia," a late stage of which is represented by Banti's disease, where the long-continued portal irritation has resulted in cirrhosis of the liver. It was thought, however, that supposing the case to be one of splenic anæmia, the tremendous size of the spleen could only mean a very advanced stage of the condition in which hepatic involvement must already have occurred ; yet evidence of cirrhosis was entirely lacking. Splenic anæmia was therefore thought to be an unlikely diagnosis.

None of the suggestions outlined above appeared to be in agreement with the clinical and pathological features of the case, and the possibility of new growth had therefore to be considered. There were two reasons for which this diagnosis was considered likely. One was the nodular character of the surface of the spleen revealed on palpation. The other was the quite remarkable diminution in the size of the organ following radium application. In all 110 milligrams of radium were applied over the tumour for a period of eight hours ; within a few days the spleen was so reduced in size that its lower pole could be felt no more than a hand's breadth below the left costal margin.

Operation was accordingly thought advisable, and splenectomy was performed by Mr. Moore on 14th February. The spleen was adherent to the liver and slightly to the diaphragm, but was removed without difficulty. Hemorrhage was negligible.

At operation no evidence was found of cirrhosis or of ascites, and no secondary deposits of growth were discovered in the abdomen. The patient made a good recovery, and for a time, although the anæmia persisted, she appeared to be doing well. Later, however, she developed a pericardial and left pleural effusion, and chest signs which were thought on both clinical and radiological grounds to indicate metastatic deposits in both lungs. Unfortunately, the patient has since died outside the Hospital, and no post-mortem examination could be obtained to confirm this observation. As to the neoplastic character of the spleen itself there could, however, be little doubt.

*Morbid Anatomy and Histology.* The spleen on removal was a considerably enlarged organ of firm consistency, weighing 34 ounces, although when the patient was first seen it must have been at least three times this size. Numerous small nodules can be seen protruding beneath the capsule (Fig. 1), and the anterior margin shows a large pale infarct evidently of quite recent formation. On section the cut surface is found studded with these nodules, which are of the same homogeneous pink colour as the surrounding splenic tissue and barely distinguishable from it except on close inspection. (Fig. 2.) The Malpighian bodies are of insignificant size and widely separated from each other.

The pink, fleshy appearance of the gross specimen is strongly suggestive of neoplastic tissue, and this diagnosis is confirmed on microscopic examination of sections removed from various parts of the organ. Histologically it is at once evident that the great size of the spleen is due entirely to a massive overgrowth of the elements of the splenic pulp ; the Malpighian bodies are few in number and atrophic, and play no part in the neoplastic process. In the pulp the endothelial structures are greatly increased ; the blood channels are large but poorly formed and lined by cells much larger and much more deeply staining than those normally present. (Fig. 3.) In places the endothelial lining is deficient, in others it is several cells thick. Scattered in large numbers through the irregular reticulum are large hyperchromatic cells, some of them of giant size, containing several closely-packed nuclei or one large vesicular nucleus staining intensely with the basic dye. (Fig. 4.) The microscopic appearance of the discrete nodules seen in the gross specimen differs little from that of the intervening pulp, except that in them there is even greater cellular

### PLATE XIII.

Figs. 1 and 2.

Surface and cut section of the spleen after removal. Multiple nodules are seen scattered beneath the capsule and through the splenic substance. The anterior border shows a large infarct caused by occlusion of a branch of the splenic artery, probably by the growth of one of these nodules in one part of its course.

Fig. 3.

Microscopic section of a portion of the splenic pulp, showing ill-formed vascular channels lined by hyperchromatic endothelial cells of large size. Similar cells are scattered irregularly through the section, completely destroying the normal architecture.

Fig. 4.

Higher power microphotograph of a portion of the splenic pulp, showing a large giant cell of obviously malignant aspect, containing a single deeply-stained vesicular nucleus. The neoplastic character of the surrounding tissue is very apparent. The lumen of the thin-walled vessel seen in the upper left-hand corner is partly filled with actively proliferating cells.

Fig. 5.

Microscopic section of one of the nodules described in the text, showing a similar general structure to that of the intervening tissue, but containing many more of the irregular multinucleated giant cells.

proliferation and the giant cells are much more numerous. (Fig. 5.) Many mitotic figures are present in all parts. The whole picture is one of sarcomatous growth arising in the endothelial cells of the splenic reticulum and producing a massive diffuse enlargement of the organ, in which the discrete nodules represent foci of particularly active growth.

Classification of the primary malignant growths of the spleen is a matter of some difficulty. Since Weichselbaum (1881) first described the condition few cases have been recorded, and Aschoff (1921) in his comprehensive text - book of pathological anatomy dismisses the subject in very few words. Ewing (1928) has collected a number of cases from the literature, and classifies them in the following three groups :—

1. Spindle - cell sarcoma: occurring as a comparatively benign circumscribed tumour, and microscopically showing spindle and round cells with trabeculæ and solid areas of fibrous tissue.

2. Endothelial sarcoma: producing as a rule multiple nodules in a greatly enlarged organ. This tumour is quite malignant, visceral metastases being commonly found. The structure consists of large irregular cells with single or multiple vesicular nuclei often with giant cell formation.

3. Primary lymphosarcoma: arising in the lymphoid cells of the splenic follicles and producing diffuse enlargement of the organ, sometimes with regional metastases. Histologically the neoplastic cells are of the type of large or small lymphocytes.

The largest of these groups is the second one, to which the case here recorded undoubtedly belongs. In this group the new growth is regarded as taking origin from the endothelium of the splenic pulp (Bunting, 1923), or from the endothelial cells of the splenic follicles (Foix and Roemmele, 1912). In the

L

present case the excessive overgrowth of the sinus endothelium and the atrophic character of the follicles indicate clearly an origin in the splenic pulp. On the other hand, in Foix and Roemmele's case, as well as in a similar case demonstrated by Professor Hadfield at a recent meeting of the Pathological Society, the Malpighian bodies were greatly enlarged and stood out with remarkable prominence on the cut surface of the organ. This group, then, would appear to contain at least two types of growth, although the endothelial origin is clear in each case.

Unfortunately, in the present case the occurrence of metastatic deposits lacks confirmation. It seems likely, however, that secondary growth took place, considering the well-recognized tendency of these tumours to metastasise and the rapid clinical course pursued in the present instance ; the patient was dead within three months of the commencement of her illness.

My best thanks are due to Dr. J. A. Birrell, Dr. Carey Coombs and Mr. Clifford Moore for kind permission to make use of their notes.

REFERENCES.

Aschoff, *Pathol. Anat.*, 1921, ii., 161.
Bunting, *Univ. Pa. Bull.*, 1903–4, 16, 188.
Ewing, *Neoplastic Diseases*, 3rd Ed., 1928, 422.
Foix and Roemmele, *Arch. med. exp.*, 1912, 24, 111.
Weichselbaum, *Virchow's Archiv.*, 1881, 85, 554.

## Figures and Tables

**Figs. 1 and 2. f1:**
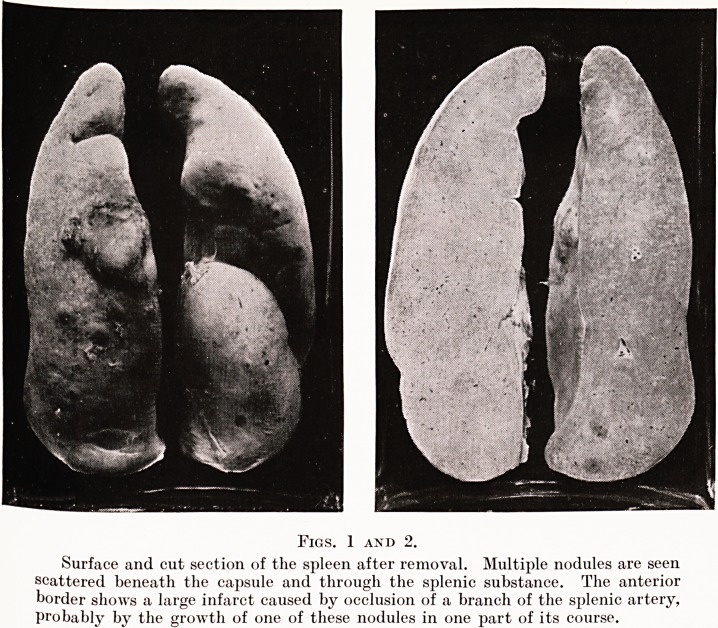


**Fig. 3. f2:**
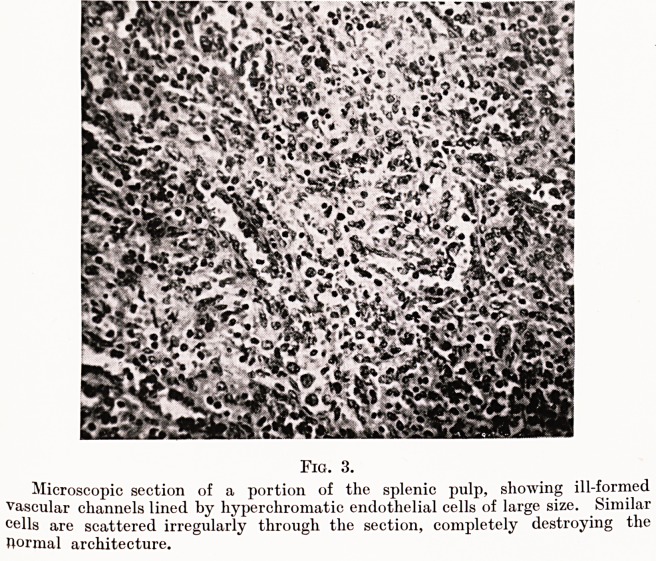


**Fig. 4. f3:**
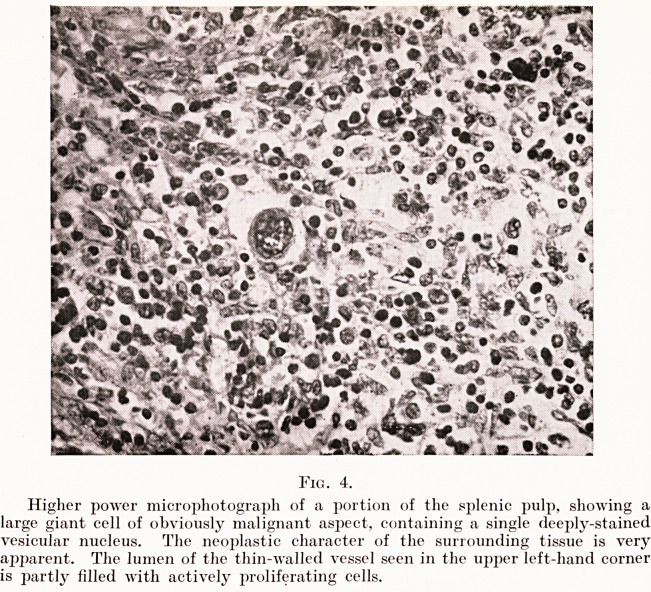


**Fig. 5. f4:**